# The Antiseptic Octenidine Inhibits Langerhans Cell Activation and Modulates Cytokine Expression upon Superficial Wounding with Tape Stripping

**DOI:** 10.1155/2019/5143635

**Published:** 2019-03-03

**Authors:** Nenad Nikolić, Philip Kienzl, Pooja Tajpara, Martin Vierhapper, Johannes Matiasek, Adelheid Elbe-Bürger

**Affiliations:** ^1^Department of Dermatology, Division of Immunology, Allergy and Infectious Diseases, Medical University of Vienna, Vienna, Austria; ^2^Department of Surgery, Division of Plastic and Reconstructive Surgery, Medical University of Vienna, Austria; ^3^Department of Plastic, Aesthetic and Reconstructive Surgery, St. Josef Hospital, Vienna, Austria

## Abstract

Ideal agents for the topical treatment of skin wounds should have antimicrobial efficacy without negative influence on wound healing. Octenidine (OCT) has become a widely used antiseptic in professional wound care, but its influence on several components of the wound healing process remains unclear. In the present study, we have used a superficial wound model using tape stripping on human full-thickness skin ex vivo to investigate the influence of OCT on epidermal Langerhans cells (LCs) and cytokine secretion pattern of skin cells during wound healing in a model without disruption of the normal skin structure. Histological and immunofluorescence studies showed that OCT neither altered human skin architecture nor the viability of skin cells upon 48 hours of culture in unwounded or wounded skin. The epidermis of explants and LCs remained morphologically intact throughout the whole culture period upon OCT treatment. OCT inhibited the upregulation of the maturation marker CD83 on LCs and prevented their emigration in wounded skin. Furthermore, OCT reduced both pro- and anti-inflammatory mediators (IL-8, IL-33, and IL-10), while angiogenesis and growth factor mediators (VEGF and TGF-*β*1) remained unchanged in skin explant cultures. Our data provide novel insights into the host response to OCT in the biologically relevant environment of viable human (wounded) skin.

## 1. Introduction

The skin is one of the body's largest interfaces and is exposed to the outer environment, functioning as a physical barrier to protect against the invasion of pathogens. In addition to mechanical defense, T cells and immature CD1a^+^CD207^+^ Langerhans cells (LCs) reside in the epidermis and participate in immunosurveillance. LCs are a specialized subset of dendritic cells (DCs) that play an essential role in sensing pathogenic microorganisms and tissue damage to initiate immune responses and maintain skin homeostasis [1–5].

In inflamed or injured skin, inflammatory signals produced by various cell types can promote LC activation and migration towards regional lymph nodes, where they elicit primary immune responses. During migration, LCs mature and upregulate the maturation marker CD83 and other molecules associated with antigen presentation [6]. LCs also play a crucial immunologic role in wound healing. Upon acute wounding, it is most important that the skin barrier function is restored as fast as possible. This is achieved by a complex wound healing process which involves four sequentially overlapping phases: hemostasis, inflammation, proliferation, and remodeling, resulting in the formation of a scar [7–10]. This well-coordinated sequence of events is regulated by a variety of cells. Immediately following wounding, DCs [11, 12], keratinocytes [13, 14], T cells [15], and mast cells [16] are activated, subsequently releasing signaling molecules to recruit other immune cells [17]. In particular, LCs represent an important immune cellular component during the initial stages of acute wound healing [18–20] and higher numbers of LCs have been shown in healing compared to nonhealing diabetic foot ulcers implying their involvement in chronic wounds as well [20]. While our understanding of LC involvement during wound healing has somewhat advanced in recent years, little is known about the influence of topically applied antiseptics on LC physiology in wound healing. In that context, a widely used molecule in modern wound care is octenidine (OCT). Compared to other antiseptics (e.g., chlorhexidine, polyhexanide, and PVP-iodine), OCT is highly effective within a short contact time at low concentrations, with a broad spectrum of antimicrobial activity against (even multidrug resistant) bacteria [21–24]. Furthermore, OCT is stable at pH 1.6-12.2, which is crucial in wound care due to pH change in the wounding process [25]. In addition, it is the only antiseptic which remains active locally for up to 48 hours and is not associated with systemic side effects [26, 27]. OCT is widely used in Europe for skin, mucous membrane, and wound antisepsis [25, 28] as well as for patient decolonization in various clinical settings [29–31], and resistances have not been reported [32]. Even though very few cases of irritant and/or allergic contact dermatitis have been reported when superficial skin infections were treated with OCT [33, 34], it is generally considered to be a safe and efficient antiseptic [25].

Results from animal studies [35–37] and clinical data [26, 38–41] have shown that besides its high antimicrobial effects, OCT may also have a positive influence on wound healing, including significant improved scar quality after abdominoplasty [42]. The treatment of chronic venous leg ulcers with OCT was associated with higher healing rates [39] and appears to have positive influence in skin transplantation in patients with impaired wound healing [38].

As animal wound repair can never be a direct and true reflection of human wound healing and its clinical challenges, it is essential to use human skin as the basis of a wound healing model because the pathology and physiology of healing is identical to that found in patients. Furthermore, there are increasing restrictions in Europe for using animals for testing properties of compounds and creams. Therefore, in line with the 3Rs (reduction, refinement, and replacement of animal models), we have used a superficial wound model using tape stripping on human full-thickness skin ex vivo as it is the simplest partial thickness injury of the skin involving removal of the *stratum corneum* leaving the epidermal compartment generally intact, thus allowing to study the effects of OCT on epidermal LCs which play an essential immunologic role during wound healing. Further, each phase of wound healing can be characterized by the secretion of cytokines, chemokines, and growth factors which were also analyzed.

## 2. Materials and Methods

### 2.1. Skin Specimens and Treatment/Culture Procedures

Skin was collected from anonymous healthy participants (aged 20-55 years) undergoing routinely performed body contouring surgeries and processed within 1-3 hours. No morphological or histological pathologies of the skin were observed. The study was approved by the ethics committee of the Medical University of Vienna and conducted according to the Declaration of Helsinki principles. Written informed consent from the participants was obtained.

To generate a superficial wound, the *stratum corneum* was removed using a standardized tape-stripping method as reported previously by our group [43]. For this, D102-squame standard self-adhesive discs (CuDerm Corporation, USA) were applied with a constant pressure for 10 seconds. Fifty consecutive tape strips were made on the identical spot by the same performer to reduce variability. The efficient removal of the *stratum corneum* was tested by immunohistochemical staining of punch biopsies (Ø = 8 mm) taken from wounded (=tape stripped) skin. In parallel, skin biopsies were cultured at the air-liquid interphase in triplicates per group in DMEM complete medium (supplemented with 10% fetal bovine serum and 1% penicillin-streptomycin (Gibco, Austria)) for 24 to 48 hours without treatment and application of 50 *μ*l control gel (Normlgel®, 0.9% *w*/*w* sodium chloride, Mölnlycke Health Care, Sweden) or 50 *μ*l OCT (octenilin® wound gel, 0.05% OCT, Schülke & Mayr GmbH, Germany) onto the epidermal side of each biopsy. Supernatants were collected at selected time points and frozen at -80°C for further analysis.

### 2.2. Histology and Epidermal Sheet Preparation

From skin explants, one-third was embedded in the optimal cutting temperature compound (Tissue-plus, Scigen Scientific Inc., USA), snap frozen in liquid nitrogen, and stored at -80°C until further processing and another third was fixed in 7.5% formaldehyde overnight and embedded in paraffin. Five *μ*m sections were cut and stained with hematoxylin and eosin to assess tissue morphology. From the last third of the biopsies, skin was incubated on 3.8% ammonium thiocyanate solution for 1 hour at 37°C (Carl Roth GmbH + Co. KG, Germany). Subsequently, the epidermis was separated from the underlying dermis, washed twice for 5 minutes with PBS, fixed with ice-cold acetone (Merck, USA) for 10 minutes, and stored at -80°C until further processing.

### 2.3. Skin Cell Apoptosis

Skin cryosections (5 *μ*m) were stained with an activation-specific anti-caspase 3 polyclonal rabbit antibody (Cell Signaling, USA) and visualized with Alexa Fluor 546 goat anti-rabbit. As a positive control, normal human skin was exposed to UVB (280–320 nm) and similarly analyzed [44].

### 2.4. Immunofluorescence

Staining of acetone-fixed epidermal sheets was performed with the following primary antibodies: CD1a (mIgG2b; BioLegend, USA), CD83 (BD Biosciences, USA), CD207 (Sigma-Aldrich, USA), and HLA-DR-Alexa88 (mIgG2; BioLegend, USA). Primary antibodies were incubated overnight at 4°C and respective isotype controls were performed. After a washing step, species- and isotype-specific secondary Abs goat anti-mouse Alexa Fluor488 and goat anti-rabbit Alexa Fluor546 (both Life Technologies, USA) were added when needed and incubated for 1 hour at room temperature. Sheets were mounted with 20 *μ*l mounting media containing DAPI (Vector Laboratories Inc., USA). Images were taken using the AX70 microscope with the imaging software MetaMorph version 7.8.6.0 (Olympus, Germany).

### 2.5. Immunohistochemistry

Acetone-fixed epidermal sheets were washed for 5 minutes with wash buffer followed by blocking of endogenous peroxidase activity for 5 minutes in methanol containing 0.03% hydrogen peroxide. Subsequently, sheets were incubated with an antibody directed against CD207 (Leica Biosystems, Germany) for 1 hour at room temperature, followed by a secondary antibody for 20 minutes at room temperature. Sheets were then incubated in streptavidin peroxidase solution for 20 minutes, and staining was visualized with amino-ethyl-carbazole (all Dako). Finally, sheets were mounted with Aquatex (Merck Millipore, USA) and examined.

### 2.6. Quantification of Cells in Skin Sections and Epidermal Sheets

**Figure 1:**
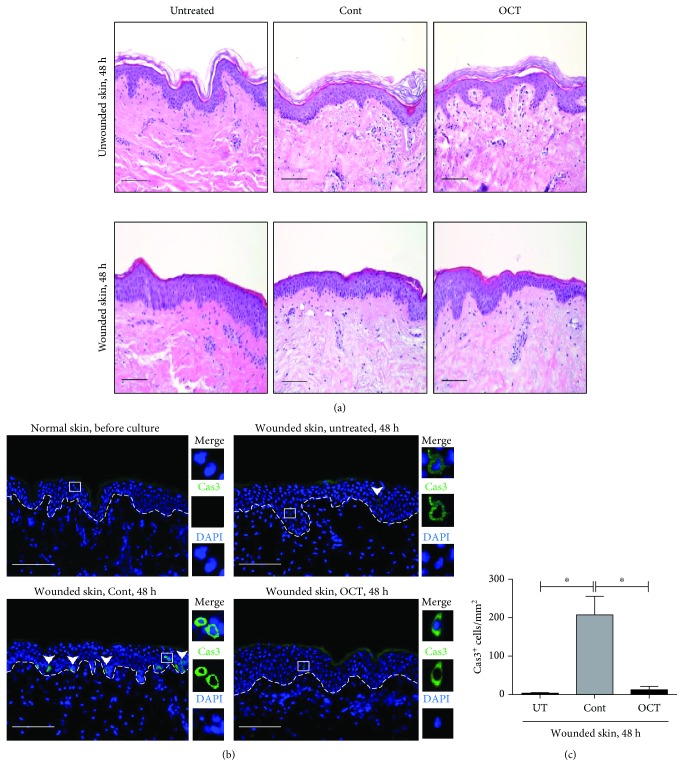
Hematoxylin and eosin-stained paraffin sections (a) and active caspase 3-stained cryosections counterstained with DAPI (nuclear stain, blue) (b) upon indicated treatments and culture are shown. Dotted line in (b) demarcates basement membrane and arrowheads denote caspase 3^+^ cells (green). One representative donor of 6 is shown. Scale bar = 100 *μ*m. Mean numbers±SD of caspase 3^+^ cells (c) are shown (*n* = 6). Unpaired *t*-test, ^∗^*P* ≤ 0.05.

**Figure 2:**
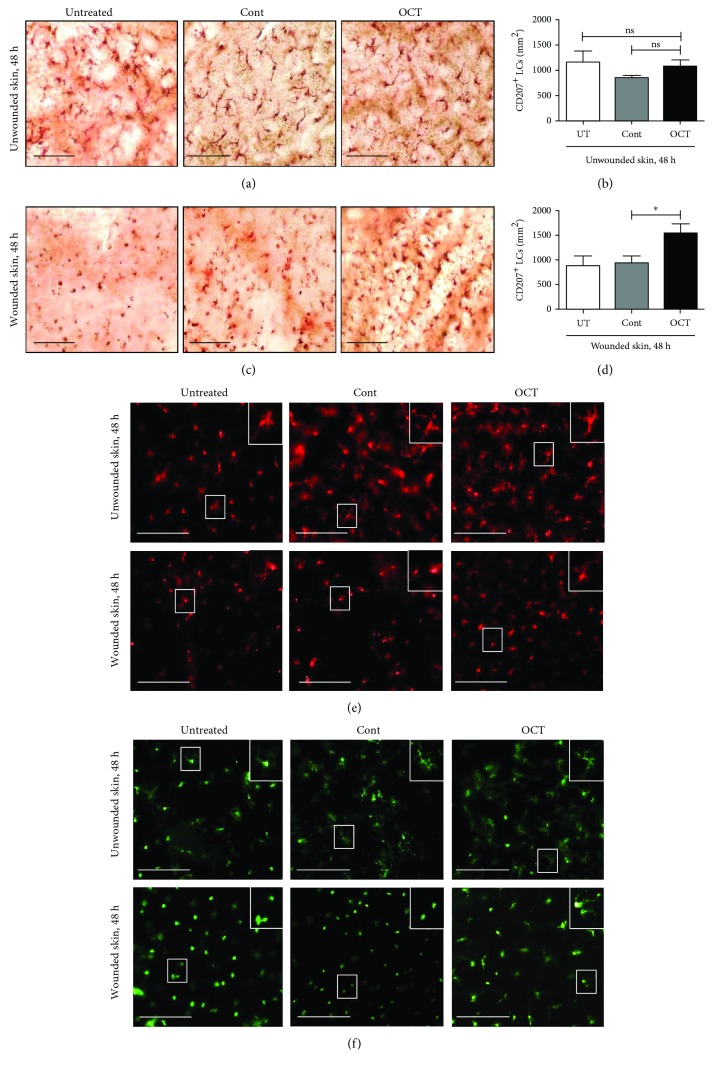
LCs stained with antibodies directed against CD207 (brown; a, c), CD1a (red; e), and HLA-DR (green; f) on epidermal sheets isolated from indicated groups and treatments. One representative donor of 7 (CD207) and of 3 (CD1a and HLA-DR) is shown. Scale bar = 100 *μ*m. Mean numbers±SD of CD207^+^ LCs (b, d) are shown (*n* = 7). Ns = not significant, unpaired *t*-test, ^∗^*P* ≤ 0.05.

Immunoreactive cells were counted in 6 images/section (Figure 1(c)) or epidermal sheets (Figures 2(b) and 2(d)) from a total of 6 different sections or epidermal sheets/donor from all experimental groups in 6 (Figure 1) or 7 (Figures 2(b) and 2(d)) different donors using ImageJ (1.51j, Wayne Rasband, National Institutes of Health, USA). Data are presented as mean±standard deviation (SD) of all measurements.

### 2.7. ELISA

96-well plates were coated with the appropriate capture antibodies: IL-8 (M801; Thermo Fisher Scientific, USA) and IL-10 (BioLegend, USA) overnight at 4°C and IL-33, VEGF, TGF-*β*1 (all R&D, USA) overnight at room temperature. On the next day, plates were washed with wash buffer (PBS-Tween 0.05%), incubated with blocking buffer (IL-8: 4% BSA in PBS-Tween 0.05%) or reagent diluent (IL-33, VEGF: 1% BSA in PBS) or block buffer (TGF-*β*1: 5% Tween-PBS) or assay diluent (IL-10) for 1 hour at room temperature. Standards and samples were applied to plates and incubated for either 1 hour (IL-8) or 2 hours (IL-33, VEGF, TGF-*β*1, and IL-10). The activation of latent TGF-*β*1 in supernatants was assessed by adding 1 N HCl for 10 minutes and stopped with 1.2 N NaOH/0.5 M HEPES. Next, the respective detection antibodies were incubated for either 1 hour (IL-8 and IL-10) or 2 hours (IL-33, VEGF, and TGF-*β*1) and subsequently incubated with a streptavidin-HRP for 20-30 minutes. TMB substrate solution (Thermo Fisher Scientific) was added and incubated for 20 minutes (IL-8, IL-33, VEGF and TGF-*β*1) or 30 minutes (IL-10) in dark at room temperature. After adding 0.18 M H_2_SO_4_ (IL-8) and 2 N H_2_SO_4_ (IL-33, VEGF, TGF-*β*1, and IL-10) to stop the reaction, the optical densities at 450 nm were measured using Multiskan™ FC Microplate Photometer (Thermo Fisher Scientific).

### 2.8. Statistical Analysis

Data was analyzed using GraphPad Prism 5 (GraphPad Software, USA). Unpaired *t*-test was used for comparing means. The results were considered significant with *P* values smaller than 0.05.

## 3. Results

### 3.1. OCT Neither Alters Skin Anatomy Nor Enhances Apoptosis in Skin Cells upon Wounding

To test whether the removal of the *stratum corneum* may influence the penetration capacity of topically applied OCT and consequently affect morphological and behavioral changes of skin cells when compared with unwounded OCT-treated skin, a human full-thickness skin ex vivo culture model was employed. We comparatively assessed unwounded with wounded human skin explants after culture without or with topical application of OCT or control gel. Compared with unwounded human skin, OCT did not cause obvious changes in the skin structure in wounded skin within 48 hours (Figure 1(a)). Thus, OCT does not alter the human skin architecture and preserves the structure of the epidermis and dermis. Next, we analyzed whether OCT induces apoptosis of skin cells in cultured wounded skin. Similar to untreated normal skin, we found no caspase 3^+^ cells in wounded skin before culture (data not shown), whereas caspase 3 activation was generally detected in some epidermal cells of all three groups and was most pronounced upon application of the control gel (Figure 1(b)). Quantitative analysis revealed significantly higher numbers of caspase 3^+^ cells in cryosections derived from control gel treatment compared to OCT and untreated groups when assessed 48 hours upon culture (Figure 1(c)). These observations suggest an increased apoptosis due to the wounding procedure itself and subsequent application of control gel rather than OCT treatment.

### 3.2. OCT Preserves LC Morphology and Prevents their Emigration and Maturation upon Wounding

**Figure 3:**
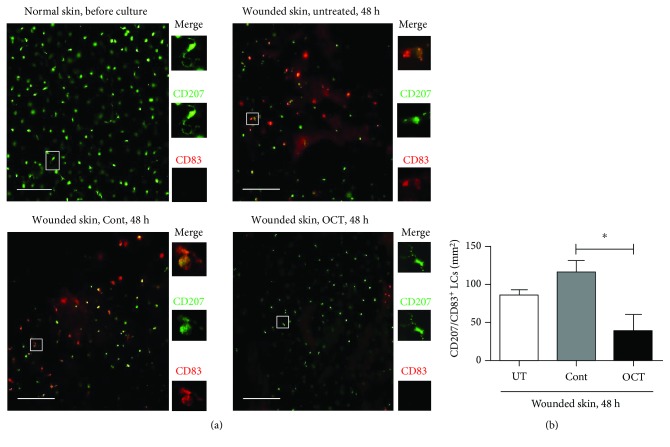
CD83^+^ (red) and CD207^+^ (green) LCs (a) in epidermal sheets isolated from indicated groups and treatments are demonstrated. One representative donor of 7 is shown. Scale bar = 100 *μ*m. Numbers of CD83^+^CD207^+^LCs (b) are shown (*n* = 7). Unpaired *t*-test, ^∗^*P* ≤ 0.05.

Once human skin is excised and subsequently cultured, epidermal LCs get activated and start to emigrate from the epidermis [6]. This nonantigen-mediated reduction of the LC density in the epidermis is visible in normal human skin specimens upon culture and was used as a baseline against which LC changes (frequency and mobilization) in skin samples in response to topical application of OCT or control gel. Analysis of freshly isolated, unwounded, and untreated epidermal sheets before culture that were stained with an antibody directed against CD207 revealed a network of highly dendritic LCs (data not shown) as previously reported [3]. LCs also exhibited many dendrites in untreated as well as OCT or control gel-treated unwounded skin after 48 hours of culture as evidenced by staining of epidermal sheets with antibodies directed against CD207, CD1a, and HLA-DR (Figures 2(a), 2(e), and 2(f); upper panels). Of note, we found a trend toward slightly less LCs in general and fewer dendrites per LCs in particular compared to freshly isolated unwounded skin (data not shown). No significant changes in LC density became apparent in untreated unwounded skin compared to unwounded skin upon topical application of OCT or control gel (Figure 2(b)). However, when the skin was wounded, LCs underwent distinct changes after 48 hours of culture in all groups. Many cells appeared round, some cells had only short surface protrusions or formed one or two single dendrites (Figures 2(c), 2(e), and 2(f); lower panels and insets). Unexpectedly, significantly more LCs were present with a better preservation of the dendritic morphology in the OCT group compared to the control group (Figures 2(c), 2(e), and (f), lower panels and insets). We next investigated whether this observation may correlate with an inhibition of LC maturation. In freshly isolated, unwounded skin, CD207^+^ LCs did not express CD83 (Figure 3(a)). However, after wounding and culture for 48 hours, many CD207^+^CD83^+^ LCs were found in epidermal sheets from untreated and control gel-treated skin, while only some double-positive LCs were found in OCT-treated epidermis (Figure 3(a)). Subsequent enumeration revealed significantly lower numbers of CD207^+^CD83^+^ LCs in epidermal sheets of OCT-treated wounded skin compared to control gel (Figure 3(b)). Our observation that OCT prevents the emigration and maturation of LCs in wounded but not unwounded skin suggests that a potentially higher concentration of OCT in wounded skin may regulate inflammatory cytokines/factors/receptors related with LC maturation and migration as well as/or cytokines and factors that are crucial in balancing/resolving inflammatory responses in our skin model.

### 3.3. OCT Significantly Inhibits the Secretion of IL-8, IL-33, and IL-10 but Not VEGF and TGF-*β*1

**Figure 4:**
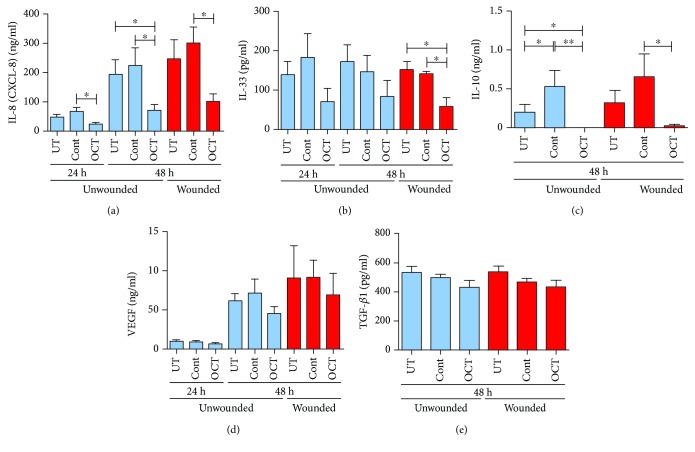
Secretion levels of the indicated cytokines were quantitatively determined by analyzing skin explant supernatants with ELISA. Data are mean±SD (*n* = 6). Unpaired *t*-test, ^∗^*P* ≤ 0.05, ^∗∗^*P* ≤ 0.01. UT: untreated skin; Cont: Normlgel®; OCT: octenilin gel®.

Upon wounding, keratinocytes act as immunomodulators, managing inflammation via a rigorously coordinated network of inflammatory cascades, triggered by keratinocyte-receptor communication with the surroundings in a paracrine and autocrine manner. Among several cytokines, IL-1*β* and TNF-*α* are the principal cytokines involved in inflammation-induced LC migration. However, OCT treatment of tape-stripped skin compared to control skin revealed no significant regulation of these cytokines at the mRNA level (data not shown), implying no involvement in the LC behavior in our model. The interplay among proinflammatory and anti-inflammatory cytokines and growth factors and angiogenesis factors determines the inflammatory response. To unravel whether OCT alters their secretion, supernatants from untreated, control gel, and OCT-treated explant cultures with unwounded and wounded skin were analyzed. We found significantly lower IL-8 levels in supernatants of unwounded and wounded OCT-treated cultures compared to controls throughout the observation period of 48 hours (Figure 4(a)). Similarly, when the skin was left unwounded, there was a trend to lower IL-33 levels in OCT-treated skin explants compared to controls, which became even more apparent and statistically significant 48 hours after wounding (Figure 4(b)). Significantly lower levels of IL-10 were measured in supernatants with OCT-treated skin cultures compared to controls upon 48 hours after wounding (Figure 4(c)). Our results clearly show that OCT has either direct or indirect anti-inflammatory properties. We next investigated, whether VEGF, one of the key regulators of angiogenesis, is affected by OCT. We found very low VEGF levels in supernatants of all three groups after 24 hours of culture, which increased at 48 hours, however, with no significant difference between the various treatments groups (Figure 4(d)). Similarly, the production of TGF-*β*1, which represents one of the most important growth factors with regard to pathological scar formation during wound healing, was not significantly affected upon OCT treatment after 48 hours of culture (Figure 4(e)). Thus, VEGF and TGF-*β*1 were not altered by the topical application of OCT.

## 4. Discussion

The application of topical antiseptics and antibiotics represents the first strategy of preventing and treating wound infection. However, during the last decades, the unrestrictive use of antibiotics has led to the development of multidrug-resistant pathogens [45], while antiseptics are less likely to cause resistance because of their unspecific mode of action [28]. Data obtained in animal [35–37] and clinical [26, 38–42] studies led to the hypothesis that besides its high antimicrobial effects, OCT may also positively influence wound healing processes including better scar quality.

*Ex vivo* skin culture models have been used previously to investigate the function of the skin immune system [46, 47]. We provide further work investigating skin viability and morphology as well as following LC behavioral changes upon application of OCT. No obvious changes in skin morphology due to its application on wounded skin was detected, when the most upper layer of the skin, the *stratum corneum*, was removed to mimic a mild wound process. Even though this skin model is the most advanced to date, we are aware of its limitations since both lymph and blood vasculatures are absent, which are essential for trafficking of immune cells during tissue repair and skin disease.

Several reports have described the properties of LCs in skin organ culture [48–50] as they conceivably represent the closest laboratory model attainable to the *in vivo* environment with regard of fidelity to physiology as well as biological complexity, even though tissue viability in general vanishes from the time of excision [51]. When healthy human skin is excised and cultured, like in our experiments, LCs start to migrate from the epidermis due to mechanical trauma, triggering a degree of inflammation, orchestrated by skin cytokines, thus mimicking the first part of sensitization and innate immunity. When following LC behavior in all groups of unwounded skin, LCs only partially retracted their dendrites over the whole observation period which was most obvious when viewed in epidermal sheets at the end of the culture period. In contrast, when epidermal sheets from wounded skin were inspected, LCs in all groups displayed a “rounded” morphology already at 48 hours of culture. Previous studies demonstrated comparable changes in LCs after intradermal vaccination, showing a “rounded” morphology and lower LC numbers after 72 hours of culture [52]. Our observations about morphologic changes of LCs in wounded skin in all experimental groups correspond to previous findings [53]. A slightly more pronounced reduction in dendrites per LC was observed in control groups when compared with OCT treatment at 48 hours in wounded skin, implying that OCT may preserve the LC morphology. In line with this, LC numbers in OCT-treated wounded skin were higher compared to untreated skin or control gel. Intriguingly, higher LC numbers in the wounded OCT-treated skin also correlated with the failure of an upregulation of the maturation marker CD83 on LC when compared with the control groups implying that LCs do not undergo a maturation process. These results showed that OCT prevents LC migration to the dermis and inhibits their maturation indicating that OCT may have some influence on signals usually favoring their emigration. To address this hypothesis, potential changes in the production of cytokines and other factors were analyzed in skin explant culture supernatants. Evaluation of the proinflammatory cytokine IL-8 revealed slightly higher levels in wounded skin compared with unwounded skin at 48 hours of culture, indicating that IL-8 secretion is upregulated in wounded skin. Surprisingly, significantly lower IL-8 concentrations were identified in OCT-treated skin cultures compared to controls at all time points. It has been reported that the inhibition of mast cell activation and degranulation led to the downregulation of IL-1*β* and IL-8 in wounds, which influenced the healing response, characterized by the reduction in wound scar width and improved collagen fiber organization [54]. Previous experiments also revealed increased IL-8 levels in fibroblasts from keloid scars compared with normal human fibroblasts. This highlights a possible role of IL-8 in activation in keloid scars and leukocyte recruitment [55]. Furthermore, it has been shown that the equilibrium in inflammation regulated by low expression of proinflammatory cytokines like IL-8 is crucial in preventing the scar formation in the fetus [56]. Therefore, downregulation of IL-8 secretion by skin cells represents a key point in wound care which might explain the OCT-mediated improved outcome in scar quality as seen in clinical settings.

When analyzing other inflammatory-related cytokines, we identified that OCT largely inhibited IL-33 secretion at all time points in wounded and unwounded skin. Our findings that OCT has anti-inflammatory capacities are in line with results from previous research studies, showing that OCT prevented TNF-*α* secretion [57], a cytokine involved in inflammation, apoptosis, and immune response [58, 59]. In addition, it has been observed that OCT led to a faster decay of wound inflammation *in vivo* without occlusive cover in pigs, where redness and swelling of the wound was absent after 4 days of treatment [60]. OCT was also highly effective in the treatment of facial acne lesions [61].

A previous study investigating the correlation between IL-8 and VEGF secretion reported a possible mechanism by which IL-8 and other inflammatory mediators may promote the expression of VEGF in endothelial cells [62]. We identified similar VEGF production but significant inhibition of IL-8 secretion in OCT-treated skin. These data imply that OCT does not affect VEGF secretion but might dampen the inflammatory immune response. IL-10, one of the most important anti-inflammatory cytokines besides TGF-*β* and IL-35 [63], and also a regulatory cytokine with important functions in the control of inflammation and immune-mediated tissue damage [64], was significantly blocked after OCT treatment. Previous results have shown an increased level of IL-10 in wounded skin of mice together with high levels of TNF-*α* and more inflammation expressed by increased levels of macrophages and mast cells in wounds suggesting a controlled inflammatory process that favored successful wound healing [65]. TGF-*β*1, a promising target for the modulation of the cutaneous scarring response during wound healing that influences angiogenesis, inflammatory response, reepithelialization, and extracellular matrix remodeling and deposition, was not significantly altered after OCT treatment. These data imply that OCT does not affect TGF-*β*1 and VEGF secretion. In conclusion, we have shown that the cytokine secretion pattern of skin cells in wounded and unwounded skin upon topical OCT treatment appears to be rather similarly regulated with regard to the investigated cytokines. However, the observation that the maturation marker CD83 is not upregulated and that LCs do not emigrate from wounded skin but only in unwounded skin strongly suggests that OCT also affects other, possibly yet unexplored, cytokines/factors/receptors in wounded skin and remains to be further explored in a future study.

A reliable skin model, which recapitulates all features of human wound repair, is essential for the clinical and mechanical investigation of human cutaneous wound healing. Although providing highly relevant and promising data in a first stage, we are aware that our currently used ex vivo wound model with sequential tape stripping on human full-thickness skin has limitations, especially as it bears no relevance to deeper wound pathology. Thus, we now aim to investigate the influence of OCT in optimized, clinically even more relevant wound models (e.g., suction blister and biopsy punch) to follow molecular changes during the wound healing process in more detail for a prolonged treatment period.

In conclusion, our data not only provide novel insights into the host response to OCT within the viable human (wounded) skin but also suggest, in addition to its known antimicrobial activity, that a modulation of mediator expression might positively contribute to its wound healing influence resulting in better scar quality.

## Data Availability

The data used to support the findings of this study are available from the corresponding author upon request.
